# Crystal Structure of *Cryptosporidium parvum* Pyruvate Kinase

**DOI:** 10.1371/journal.pone.0046875

**Published:** 2012-10-09

**Authors:** William J. Cook, Olga Senkovich, Khadijah Aleem, Debasish Chattopadhyay

**Affiliations:** 1 Department of Pathology, University of Alabama at Birmingham, Birmingham, Alabama, United States of America; 2 Center for Biophysical Sciences and Engineering, University of Alabama at Birmingham, Birmingham, Alabama, United States of America; 3 Ronald E. McNair Scholar Program, University of Alabama at Birmingham, Birmingham, Alabama, United States of America; 4 Department of Medicine, University of Alabama at Birmingham, Birmingham, Alabama, United States of America; Helmholtz Centre for Infection Research, Germany

## Abstract

Pyruvate kinase plays a critical role in cellular metabolism of glucose by serving as a major regulator of glycolysis. This tetrameric enzyme is allosterically regulated by different effector molecules, mainly phosphosugars. In response to binding of effector molecules and substrates, significant structural changes have been identified in various pyruvate kinase structures. Pyruvate kinase of *Cryptosporidium parvum* is exceptional among known enzymes of protozoan origin in that it exhibits no allosteric property in the presence of commonly known effector molecules. The crystal structure of pyruvate kinase from *C. parvum* has been solved by molecular replacement techniques and refined to 2.5 Å resolution. In the active site a glycerol molecule is located near the γ-phosphate site of ATP, and the protein structure displays a partially closed active site. However, unlike other structures where the active site is closed, the α6' helix in *C. parvum* pyruvate kinase unwinds and assumes an extended conformation. In the crystal structure a sulfate ion is found at a site that is occupied by a phosphate of the effector molecule in many pyruvate kinase structures. A new feature of the *C. parvum* pyruvate kinase structure is the presence of a disulfide bond cross-linking the two monomers in the asymmetric unit. The disulfide bond is formed between cysteine residue 26 in the short N-helix of one monomer with cysteine residue 312 in a long helix (residues 303–320) of the second monomer at the interface of these monomers. Both cysteine residues are unique to *C. parvum*, and the disulfide bond remained intact in a reduced environment. However, the significance of this bond, if any, remains unknown at this time.

## Introduction

The protozoan parasite *Cryptosporidium parvum*, one of the causative agents of human cryptosporidiosis, belongs to the Coccidia subgroup in the phylum apicomplexa and causes waterborne diseases worldwide [1], [2]. Cryptosporidium oocysts can withstand common water treatment methods, including chlorination, and major outbreaks of cryptosporidiosis caused by contamination of drinking water have been reported [3], [4]. Although in healthy adults Cryptosporidium infection results in self limited diarrhea, an outbreak in 1993 in Milwaukee, WI affected an estimated 400,000 people [5]. Moreover, as an opportunistic infection it can be fatal in immunocompromised individuals, such as persons infected with HIV, especially those without access to highly active antiretroviral therapy [6].

Despite the global spread of the parasite, therapeutic options for effective treatment of *C*. *parvum* infection are limited [7]. Very few potential drug targets are known, because metabolic pathways and regulatory molecules that are key to the survival of the parasite are largely uncharacterized [8]. Therefore, expanding our knowledge of major biochemical pathways that are known to be important in protozoa, such as glycolysis, is of considerable interest [9], [10].

Early studies suggested that *C. parvum* depends heavily on glycolysis for energy [11], [12]. All of the enzymes in the glycolytic pathway were found in the cytoplasmic fraction of the oocysts [12]. Subsequently, genome sequencing revealed genes encoding all glycolytic enzymes, but genes encoding the Krebs cycle enzymes and the components of the mitochondrial complexes I to IV were missing. The lack of functional mitochondria further underscores the reliance of the organism on glycolysis [13]. However, biochemical and structural studies of glycolytic enzymes of *C. parvum* are quite limited. In contrast, the glycolytic pathway has been studied in considerable detail in trypanosomatid parasites [10], [14], and two enzymes in particular, glyceraldehyde 3-phosphate dehydrogenase and pyruvate kinase (PyK), have been targeted for designing antitrypanosomal drugs [15], [16]. More recently, PyK has been shown to be an effective target for antibacterial agents against methicillin resistant *Staphylococcus aureus* [17], [18].

PyK catalyzes the last step of glycolysis, in which the phosphoryl group of phosphoenolpyruvate (PEP) is transferred to ADP to form pyruvate and ATP, and serves as a major regulator of glycolysis [9]. There are some notable differences among PyKs from various species. In mammals four PyK isozymes are expressed, but Trypanosomes encode only one, [19], [20], [21]. In at least two members of apicomplexa, *Toxoplasma gondii* and *Plasmodium falciparum*, a second PyK localized in the apicoplast has been discovered in addition to the cytoplasmic form [22], [23]. However, only the cytoplasmic form has been identified in *C. parvum* (http://cryptodb.org/cryptodb/).

A hallmark of PyK is the allosteric regulation of its activity by various phosphorylated sugars [24]. PyKs from various organisms use different effector molecules for regulation. For example, mammalian enzymes are strongly regulated by fructose 1, 6-bisphosphate (F-1,6 BP), but trypanosomal PyK remains relatively unaffected by F-1,6 BP and is activated by submicromolar concentrations of fructose 2, 6-bisphosphate (F-2,6 BP) [20]. Cytoplasmic PyK of *T. gondii* is activated by glucose 6-phosphate and F-1,6 BP [11], [25]. Notably, *C. parvum* PyK (CpPyK) is exceptional, as it showed no allosteric property [11]. Phosphosugars, including glucose 1-phosphate, glucose 6-phosphate, fructose 6-phosphate, ribose 5-phosphate, F-1,6 BP and F-2,6BP, had no effect on the enzyme activity [11]. The only other known PyK that lacks allosteric activity is the mammalian muscle isozyme M_1_ [26].

Three-dimensional structures of PyK from mammalian, bacterial and parasitic organisms have been reported [20], [24], [27], [28], [29]. Among the parasitic proteins, *L. mexicana* pyruvate kinase (LmPyK) is the most thoroughly studied (13 out of 18 entries in the protein data bank), and structural consequences of binding substrates and allosteric regulators have been established from a series of crystal structures [20], [28], [30]. Crystal structures of PyK from three other parasites, *T. gondii*, *Trypanosoma cruzi* and *P. falciparum* (PDBID: 3KHD; unpublished) have also been reported [25], [31]. These studies demonstrated that the structures of PyKs are influenced by the presence or absence of ligands at specific sites in the protein and by the crystallization conditions [25], [30]. Recently an unpublished crystal structure of CpPyK was deposited in the protein data bank (PDBID: 3MA8). We have independently determined the structure using crystals grown under conditions significantly different from those reported for 3MA8. Here we describe the CpPyK structure and compare it with structures of PyK from other organisms and the one reported in 3MA8.

## Results and Discussion

CpPyK used for crystallographic study contained amino acid residues 2–526 and 14 additional amino terminal residues derived from the expression vector, which includes a T7-tag. The calculated molecular weight for a monomer of this CpPyK construct is 57.67 kDa. In a previous study we reported that the oligomeric state of CpPyK could not be confirmed based on the results of size exclusion chromatography [32]. However, the majority of PyKs are tetrameric, and CpPyK also displays a tetrameric assembly in the crystal structure. Examination of a partially purified protein preparation on an analytical gel filtration column showed that the elution volume of the major peak fraction containing CpPyK was similar to that of purified Catalase (Mr 232 kDa). Chromatograms showing the elution profiles are presented in Figs. S1A and S1B. SDS-PAGE analysis of the fractions (Fig. S1C) indicates that CpPyK eluted at a volume (9.5–12 ml) expected for a tetramer. Thus CpPyK exists mainly as a tetrameric protein but may remain in equilibrium with other oligomeric states.

Although detailed kinetic analysis was not performed, we confirmed that the purified protein was enzymatically active in the pH range 5.5–7.5. Initial reaction velocity was measured using conditions described for similar enzymes (see Materials and Methods), and the V_max_ of approximately 0.04 mM NADH/min was in the same range as reported for *T. gondii* PyK (Figs. S2A and S2B). It should be noted that the conditions used in our assay may not represent the optimal conditions for CpPyK activity. Enzyme activity remained unchanged in the presence of 1 mM tris(2-carboxyethyl)phosphine (TCEP), a strong reducing agent that is relatively resistant to oxidation, and up to 5 mM dithiothreitol (DTT) (Fig. S2B).

### General Description and Quality of CpPyK Structure

**Figure 1:**
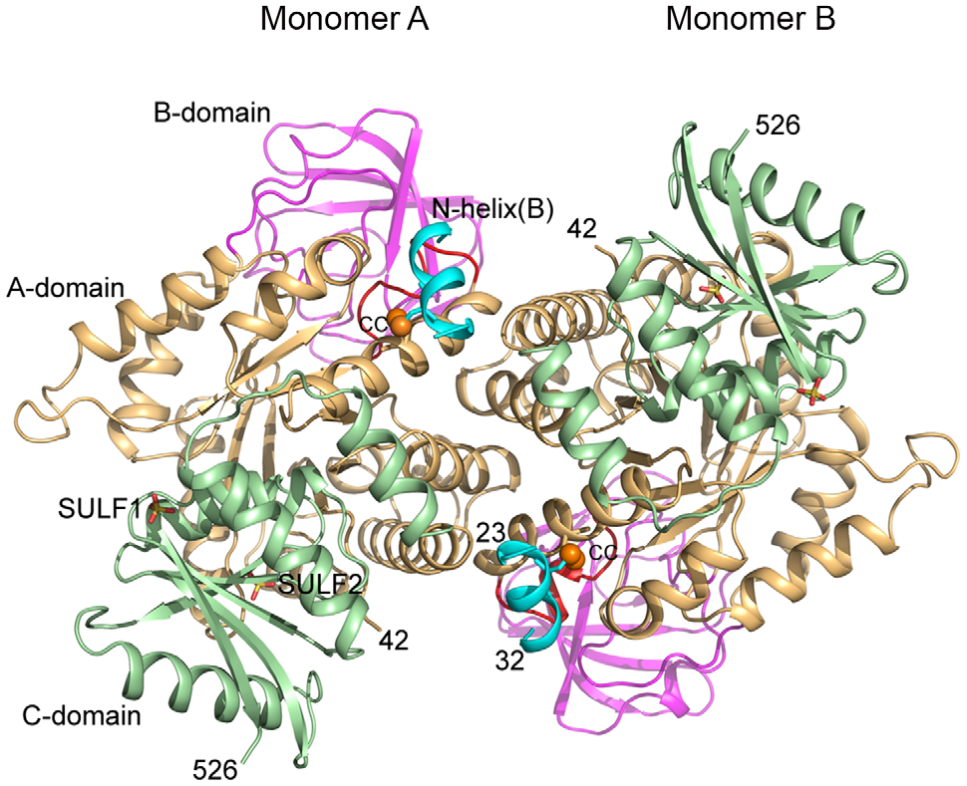
CpPyK asymmetric unit. Monomers A and B comprise the asymmetric unit and are related by a noncrystallographic 2-fold axis perpendicular to the plane of the paper. The domains in each monomer are colored as follows: N - cyan (residues 23–32), A - wheat (residues 42–112 and 212–389), B - magenta (residues 113–211), and C - light green (residues 390–526). The sulfate ions are shown as stick models; the sulfate ion bound at the effector site in each monomer is labeled SULF1. The sulfur atoms of cysteine residues 26 and 312 in each monomer are shown as orange balls; the disulfide is indicated by CC. The unwound helix α6’ is shown in red in both monomers. The A domains from these monomers form the major protein-protein interface in the tetramer.

**Figure 2:**
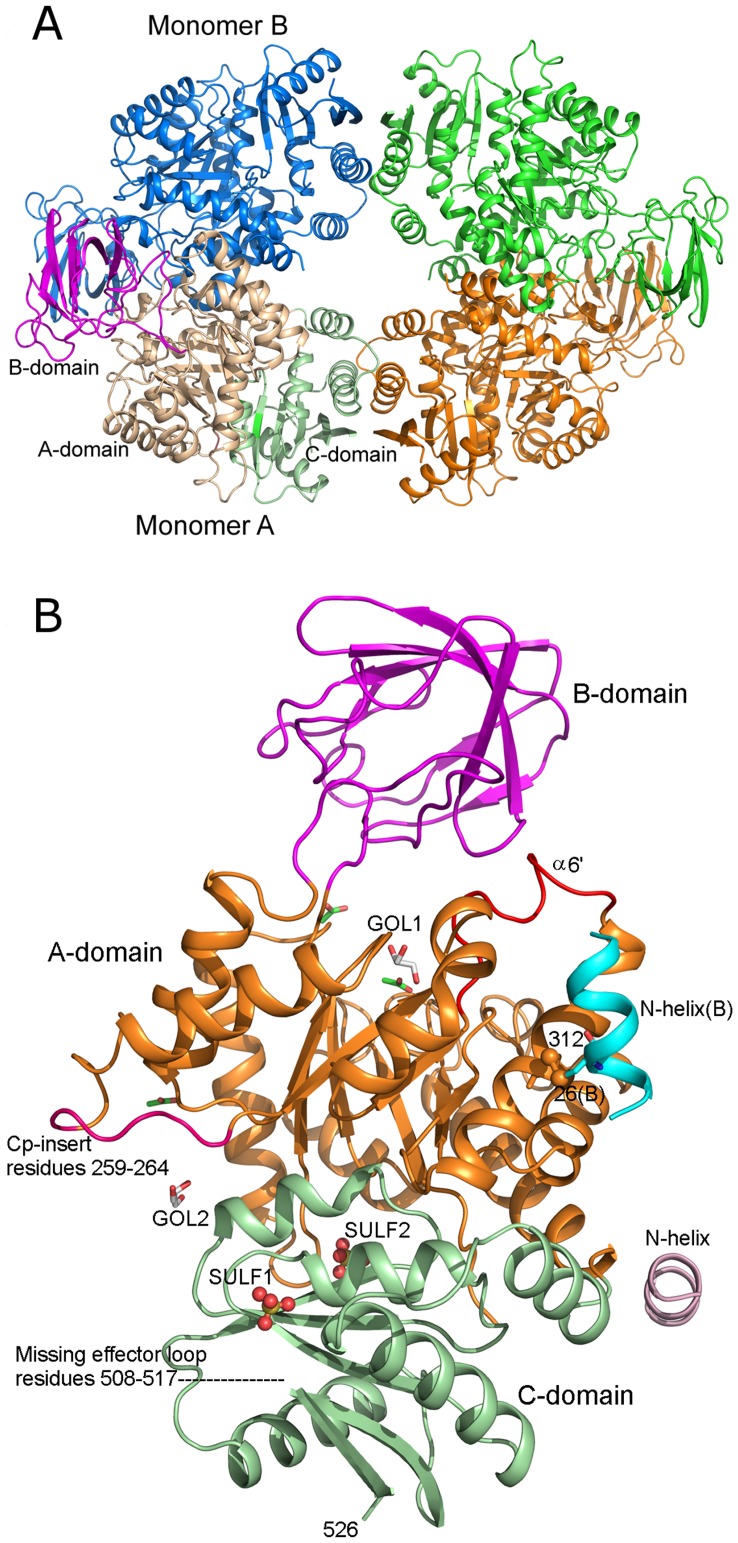
CpPyk tetramer and monomer. (A) The tetramer is generated by a crystallographic 2-fold axis. Domains of monomer A are colored the same as in Fig. 1. Symmetry related monomers are shown in green and orange. A minor interface is formed by the C-domains of the symmetry partners. (B) Monomer A. The domains are colored as follows: N - light pink, A - orange, B - magenta, C - light green. The two sulfate ions are labeled SULF1 and SULF2. The N-helix of the B monomer (cyan) is included in order to show the disulfide bond. The sulfur atoms in the disulfide bond between cysteine residues 26 and 312 are shown as orange balls. Glycerol and acetate ions are shown as stick models. The unwound helix α6’ is shown in red. The location of the missing effector loop is indicated. The loop representing the Cryptosporidium-specific insertion in the primary sequence is also labeled.

The asymmetric unit in the crystal structure contains two monomers (A & B) of CpPyK related by non-crystallographic two-fold symmetry (Fig. 1). The complete tetramer is formed with their symmetry partners related by the crystallographic 2-fold axis along the c axis (Fig. 2A). The root mean square deviation (r.m.s.d.) between monomers A and B is 0.38 Å for all 485 Cα atoms. The final model includes residues 23–32, 42–507 and 518–526 for each chain. The arrangement of the monomers in the tetramer is similar to that seen in other PyKs. The two monomers in the asymmetric unit form the major interface and bury approximately 2000 Å^2^ of surface area.

Each CpPyK molecule consists of four domains: N (residues 23–32), A (42–112 and 212–389), B (113–211) and C (390–526). The A-domain constitutes the central part of the molecule and forms a parallel (α/β)_8_ barrel (Fig. 2B). The B-domain contains nine β strands that form an antiparallel β-barrel. The active site is located at the interface of the A and B domains, and residues from both domains participate in substrate binding. The C-domain is composed of five β strands surrounded by five α-helices. The allosteric site for binding the effector molecule is located in the C-domain. The A domains of the two monomers A and B form the major interface (also referred to as large interface or A-A interface). The C-domains of monomers related by the crystallographic 2-fold symmetry form a smaller interface called C-C interface (Fig. 2A). The N domain is mostly disordered in PyK structures; the only portion visible in this structure is a short α helix referred to as N-helix (residues 23–32; Fig. 2B). Although the electron density for the polypeptide chain connecting the N-helix to the A-domain (residues 33–41) was missing, the helix for each monomer could be unambiguously assigned to the respective monomer (Figs. 1 and 2B). Furthermore, the N-helix in this structure is in a similar position as in 3MA8, in which the linker peptide was modeled. This short helix is involved in a unique interaction in CpPyK not seen in any other PyK (discussed below).

Electron density was generally excellent in the A and C domains, except for a long loop in the C-domain (residues 508–517) that was completely disordered. This loop is involved in binding of effector molecules (usually fructose biphosphates) and is typically not ordered unless the effector molecule is bound. The density was much weaker in domain B, especially for residues 118–150, 167–179, and 186–200, and the high B-factors reflect this. For monomer A the average B-factor for domains N, A and C is 52.0 Å^2^, while the average B-factor for domain B is 92.5 Å^2^. The corresponding average B-factors for monomer B are 52.5 and 109.2 Å^2^, respectively.

A summary of the data collection and refinement statistics is presented in Table 1. The overall quality of the structure of CpPyK is excellent (see Table 1 and Materials and Methods). Only Gly123 in each monomer exhibits phi, psi angles in non-allowed regions of the Ramachandran plot. Electron density for these two residues was extremely weak. The final model also contains two sulfate ions, SULF1 and SULF2 (corresponding to SO4 527 and SO4 528 in the deposited coordinates), and two glycerol molecules, GOL1 and GOL2 (GOL530 and GOL 531 in the deposited coordinates), associated with each chain. In addition there are six acetate ions and 149 water molecules (Table 2).

**Table 1:** Data-collection and refinement statistics.

Crystal data	
Space Group	P2_1_2_1_2
Unit cell parameters (Å)	a = 129.9, b = 136.9, c = 77.2
V_m_ (Å^3^ Da^−1^)	3.04
Solvent content (%)	59.6
Data collection	
Resolution range (Å)	50.0–2.50 (2.54–2.50)^a^
No. of reflections	202,725 (9168)
No. of unique reflections	47,438 (2177)
Multiplicity	4.3 (4.3)
Completeness (%)	97.8 (98.9)
R_merge_ (%)	7.1 (51.4)
Mean I/σ(I)	12.1 (1.0)
Refinement statistics	
Resolution range (Å)	49.71–2.50 (2.565–2.50)
Reflections (working set)	45,005 (3101)
Reflections (test set)	2344 (148)
R value (working set)	0.213 (0.273)
Free R value	0.249 (0.333)
No. of protein atoms	7292
No. of sulphate ions	5
No. of water molecules	149
Estimated coordinate error based on R value (Å)	0.39
Estimated coordinate error based on free R value (Å)	0.26
R.m.s. deviations from ideal values	
Bond lengths (Å)	0.005
Bond angles (°)	0.92
Mean B value (Å^2^)	61.7
Structure quality	
Ramachandran most favored (%)	96.87
Ramachandran allowed (%)	2.92
Ramachandran outliers (%)	0.21
Rotomer outliers (%)	0.24

a Values in parentheses are for the outermost resolution shell.

**Table 2:** Contacts for acetate ions.

	Residue (Atom name)	Distance (Å)
Acetate 531 (A)		
O	Gol529 (O3)	3.10
O	Asn76 (ND2)	2.90
Acetate 532 (A)		
O	His79 (ND1)	3.12
O	Arg198 (NH1)	2.32
Acetate 533 (A)		
O	Arg93 (NH1)	3.09
OXT	Arg93 (NH1)	3.16
Acetate 531 (B)		
O	Gol529 (O3)	2.64
O	Asn76 (ND2)	2.93
Acetate 532 (B)		
O	His79 (ND1)	3.32
O	Arg198 (NH1)	2.67
Acetate 533 (B)		
O	Lys404 (NZ)	3.21
OXT	Water 682	3.03

Results of primary sequence alignment using the CLUSTALW server (http://www.ebi.ac.uk/) [33], [34] show that identity between PyKs from various species varies roughly in the range 40–66% based on the evolutionary relationship between the organisms. Thus, pair-wise sequence identity between the pyruvate kinase of *C. parvum* and those of human, *E. coli*, *L. mexicana*, *P. falciparum* and *T. gondii* are 39, 42, 41, 51 and 54%, respectively, while the *P. falciparum* and *T. gondii* sequences are 66% identical. The overall architecture of CpPyK is similar to the structures of PyKs from other organisms. However, the orientation of the B-domain with respect to the A and C domains varies rather widely in various PyK structures. Therefore, pair-wise alignment of the entire molecule by superposition is influenced by the relative orientation of the B domains. For example, the r.m.s.d. between the A monomers of CpPyK and *P. falciparum* PyK is 0.86 Å, while the r.m.s.d. for the combined A and C domains only is 0.67 Å. On the other hand, the r.m.s.d. values between the A monomers of CpPyK and those of the truncated (PDBID: 3GG8) and full length versions (PDBID:3EOE) of *T. gondii* PyKs are 0.65 and 0.83 Å. When the B domain is removed from these structures the r.m.s.d. values lie around 0.6–0.7 Å. The r.m.s.d. values for the superposition of the A and C domains of CpPyK with LmPyK (PDBID: 1PKL) and human PyK (PDBID: 3GQY) are 0.76 Å and 0.85 Å, respectively.

### CpPyK Crystal Structure Contains a Disulfide Linked N-helix

**Figure 3:**
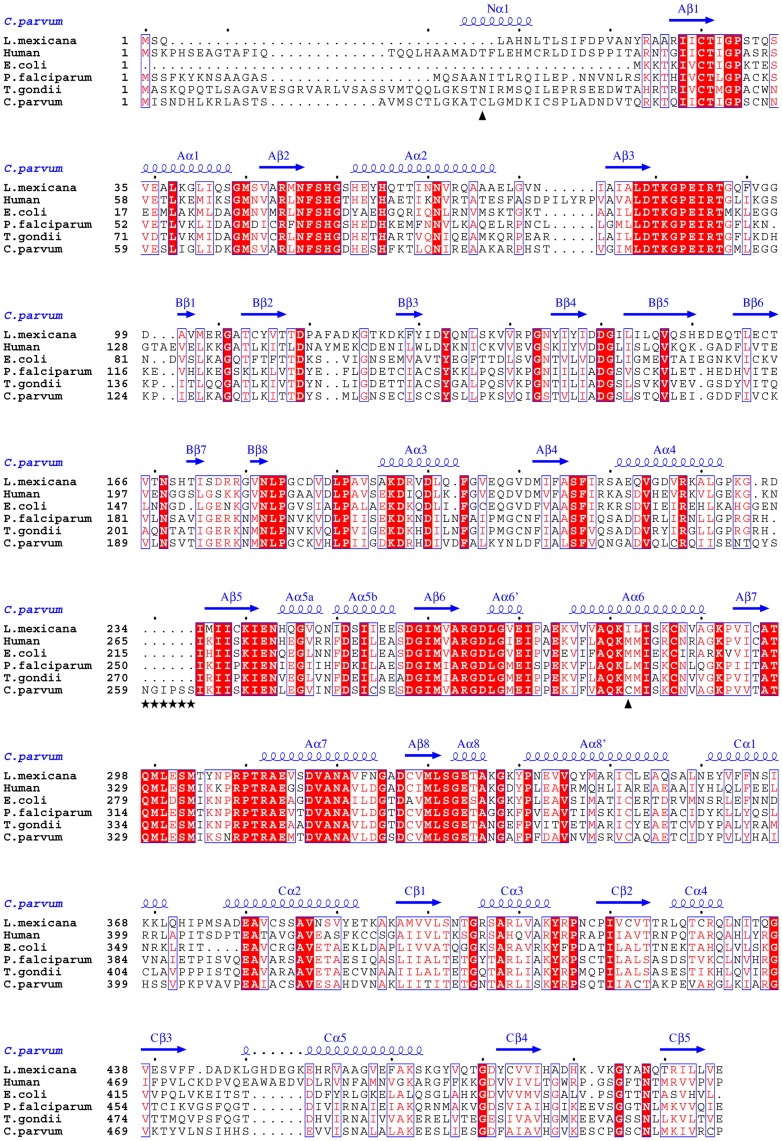
Primary sequences of pyruvate kinases from various organisms were aligned using the CLUSTALW program [33], [34]. The labeling of secondary structural elements corresponds to the CpPyK structure. The two black triangles indicate the cysteine residues involved in the disulfide bond. The stars mark the characteristic 6-residue insertion preceding the β5 strand in domain A of CpPyK.

A new feature of the CpPyK structure is that the two monomers in the asymmetric unit are linked by two right-handed disulfide bonds between Cys26 of one monomer and Cys312 of the other (Fig. 1). Analysis of the crystal structure using the SSBOND server (http://hazeslab.med.ualberta.ca/forms/ssbond.html) [35] also predicted only this pair of disulfide bonds. These two cysteine residues (out of a total of 21) are unique to Cryptosporidium (Fig. 3). Since no disulfide bond has been described in other PyK structures, and Denton et al. [11] reported that pyruvate kinase activity in *C. parvum* extract was enhanced by reducing agent, we maintained reducing conditions throughout the purification steps (see Materials and Methods). In fact, addition of reducing agents during purification was found to be necessary to prevent protein aggregation. Interestingly, although the disulfide bond appears to be exposed in the crystal structure, it was not reduced. However, any functional role of the N-domain has been ruled out, since removal of this domain had no effect on the enzymatic activity of human PyK [36]. Moreover, the structural significance of this domain is likely to be minimal, since in some crystal structures of full length PyKs a large portion of the N-domain remains disordered [25]. It should be noted that Cys312 is located in the long helix (residues 303–320) that is involved in interactions with the other monomer in the asymmetric unit across the large interface between adjacent A domains (Fig. 1) and is connected to the α6’-helix (residues 293–302), which changes conformation upon substrate binding.

### CpPyK Active Site is Partially Closed

**Figure 4:**
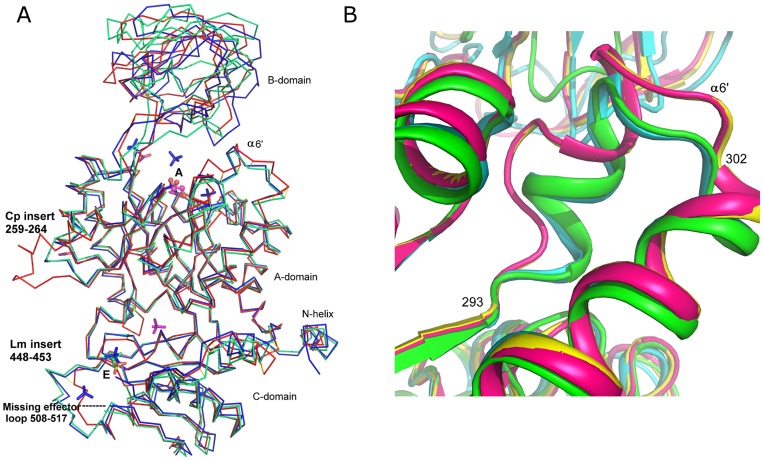
Comparison of CpPyK with LmPyK. (A) Superposition of A monomers of CpPyK (red), *L. mexicana* PyK without sulfate ion in the active site (1PKL, green), and *L. mexicana* PyK with sulfate ion in the active site (3E0V, blue). Sulfate ions bound at the effector binding site (labeled E) in all three structures are shown as stick models: CpPyK (yellow and red), 1PKL (green) and 3E0V (blue). Sulfate ions in the active site area (labeled A) in 3E0V are also shown in blue. One additional sulfate in the CpPyK structure at the interface of the C and A domains is shown in magenta. The glycerol molecule in the active site of CpPyK is shown as a ball and stick model. The two areas of the protein structures affected by the insertions in *L. mexicana* and *C. parvum* sequences are labeled. (B) Unwinding of helix α6’ in both monomers of CpPyK (red and yellow) as compared to 3E0V (cyan) and 1PKL (green). Residues 293 and 302 for CpPyK are labeled.

Specific structural changes are observed in PyKs in response to binding of substrate and effector molecules. Moreover, structural changes resulting from differences in crystallization conditions have also been reported. For example, differences in the structures of full length and truncated versions of *T. gondii* PyK have been attributed to different crystallization conditions [25]. From this consideration CpPyK crystals are quite comparable to the LmPyK crystal grown from ammonium sulfate in acidic buffer (pH 4.0–4.6) at 4°C [28]. Although these latter crystals were grown in the presence of F-1,6BP, only sulfate ions were located in the effector binding site as well as at the sites for binding PEP and ATP. Crystals of the apo form of LmPyK (without any added substrate, effector or analog) were also grown at a low pH (4.8) in the presence of ammonium sulfate. Fig. 4A shows superposition of these two LmPyK structures with the CpPyK structure. The only significant deviations in the A and C-domains of these three structures are in the two regions where there is a 6-residue insertion in the sequence. Notably, the orientation of the B-domain relative to the A-domain in the CpPyK structure is more similar to the LmPyK structure that has sulfate ions bound in the active site (PDBID: 3E0V), while in the apo-LmPyK structure the orientation is markedly different. Therefore, the active site of CpPyK appears to mimic the partially closed conformation observed in the LmPyK sulfate-bound form (PDBID: 3E0V). This LmPyK structure has two sulfate ions in the active site occupying the positions for the β and γ-phosphate groups of ATP, but the CpPyK structure contains no sulfate ion at these positions. Instead, there is a glycerol molecule (GOL1) located in the active site of CpPyK at nearly the same position occupied by the ATP γ-phosphate.

Apart from the orientation of the B-domain, the major conformational difference between these structures is in the residue range 293–302 in CpPyK (α6’ shown in red in Fig. 2B); in both LmPyK structures the corresponding region is α-helical, but in CpPyK the helix is completely unwound in the A monomer and contains only a short helical stretch in the B monomer (Fig. 4B). Comparison with the structure of human PyK showed that it is very similar to the CpPyK structure. There is no significant difference in the active site. There is an extended loop in the CpPyK structure due to a characteristic 6 residue insertion (residues 259–264; Fig. 2B, Fig. 3 and Fig. 4A) found only in Cryptosporidium sequences. This loop lies at the exterior of the molecule.

### Unwinding of α6’ Helix in the Active Site of CpPyK

**Figure 5:**
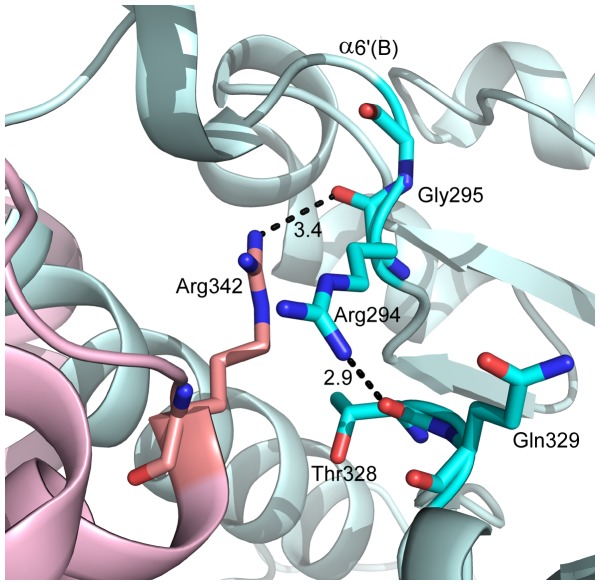
Cartoon drawing of monomers A (light pink) and B (light cyan) showing the orientation of the Arg342 side chain from monomer. A. Unwinding of the α6’ helix of monomer B results in the movement of the main chain carbonyl group of Gly295 too far away for interaction with Arg342 of monomer A. Arg294, Thr328 and Gln329 of the B monomer are also shown.

Based on the crystal structures of PyKs with and without various ligands, a model for the structural rearrangement at the active site has been proposed [28], [30]. According to this model, the transition from the inactive to active state involves a rigid body rotation of the A- and C-domains of 6° around a pivot point at the base of the αβ-barrel of domain A. Upon binding of substrates in the active site, the side chain of a conserved arginine residue (Arg310 in LmPyK, corresponding to Arg342 in CpPyK) moves into the vicinity of the active site of the adjacent subunit at the large interface, where it forms two stabilizing hydrogen bonds with backbone carbonyls of arginine and glycine residues (corresponding to Arg294 and Gly295 in CpPyK) located in the α6’ helix. This interaction across the A-A interface appears to be functionally important, since residues in the α6’ helix (^294^RGDLGME^300^ in CpPyK) are highly conserved in all PyKs, and mutation of Arg310 in LmPyK results in the loss of enzymatic activity [36]. In the present structure, even though there is no substrate, analog or sulfate ion bound at the active site, Arg342 assumes a conformation somewhat similar to that observed in substrate-bound PyK structures with its side chain pointed toward the active site of the other monomer (Fig. 5). A similar conformation is observed when sulfate ions are bound in the active site of LmPyK [28]. On the other hand, while unwinding of the α6’ helix was observed when the sulfate ions were removed from the crystals of LmPyk, in CpPyK the α6’ helix remains unwound in both monomers, and the carbonyl of Gly295 is far away from the Arg342 side chain. Instead, the Arg342 side chain forms a long hydrogen bond with the carbonyl of Arg294. Arg294 is also hydrogen bonded to the Thr328 carbonyl oxygen through its side chain. Unwinding of the α6’ helix is therefore consistent with the absence of sulfate ion from the active site [28]. Nevertheless, the CpPyK active site remained in a partially closed conformation. The GOL1 molecule at the ATP binding site in the CpPyK crystal structure, therefore, has a similar effect as the sulfate ion in the active site. In the A monomer the glycerol (GOL1) forms hydrogen bonds with the side chain nitrogen of Asn76, the side chain of Glu272 and the hydroxyl group of Ser362. In the B monomer only the Glu272 side chain is involved in hydrogen bonding with the glycerol, and additional hydrogen bonds are formed with water molecules and an acetate ion. In the activator-bound human PyK (PDBID: 3GQY) an L-tartaric acid is found in the same position.

### Sulfate Binding to CpPyK

**Figure 6:**
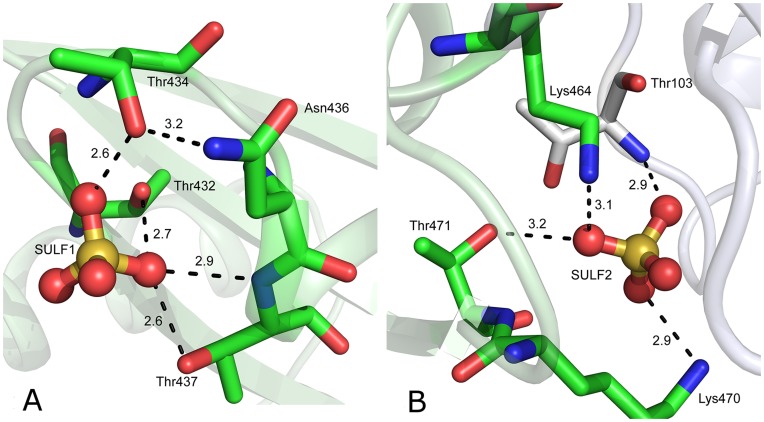
Binding of sulfate ions in CpPyK. Potential hydrogen bond donors and acceptors are indicated by dotted lines with distances in Å. (A) SULF1 and nearby residues in the allosteric site. (B) SULF2 and nearby residues.

Each monomer in the asymmetric unit of CpPyK binds two sulfate ions at equivalent positions, one in the C-domain and the other at the interface of the A and C domains (Fig. 2B). The sulfate ion in the C-domain (SULF1) occupies a position corresponding to the 6-phosphate of the effector molecule in different PyKs (location E in Fig 4A). This sulfate ion forms hydrogen bonds with hydroxyl groups from Thr432, Thr434 and Thr437, as well as one hydrogen bond with the N atom of Thr437 (Fig. 6A). The corresponding residues in other PyKs are always serine or threonine, with one exception in *E. coli*, where one of the threonine residues (Thr434 in CpPyK) is replaced by glycine. In spite of the presence of the sulfate ion, the loop between the two C-terminal β strands in the molecule (residues 508–517) is disordered as seen in other PyK structures without any effector molecule. As discussed earlier, CpPyK is not activated by fructose biphosphates or a number of other phosphosugars [11]. However, the effector site is capable of binding a sulfate ion. CpPyK may, therefore, use a different effector molecule or a different regulatory mechanism [11]. This hypothesis is further supported by the observation that in LmPyK four pairs of stabilizing salt bridge interactions between Asp482-Arg493 and Lys484-Glu498 are formed in the tetramer when F-2,6BP is bound in the effector site [30]. In CpPyK the aspartate residue in the first pair is replaced by valine (Val509), and the glutamate residue in the second pair is replaced by Pro526. Therefore, neither salt bridge would be possible in CpPyK.

The second sulfate ion (SULF2) occupies a small pocket at the interface of the A and C domains and is hydrogen-bonded to two NZ atoms (Lys464 and Lys470), the hydroxyl oxygen of Thr471 (all in domain C), and the peptide N atom of Thr103, which belongs to domain A (Fig. 6B). Three of these residues are unique to Cryptosporidium, and Thr471 is found only in another apicomplexan parasite, *T. gondii*.

### Comparison with 3MA8

**Figure 7:**
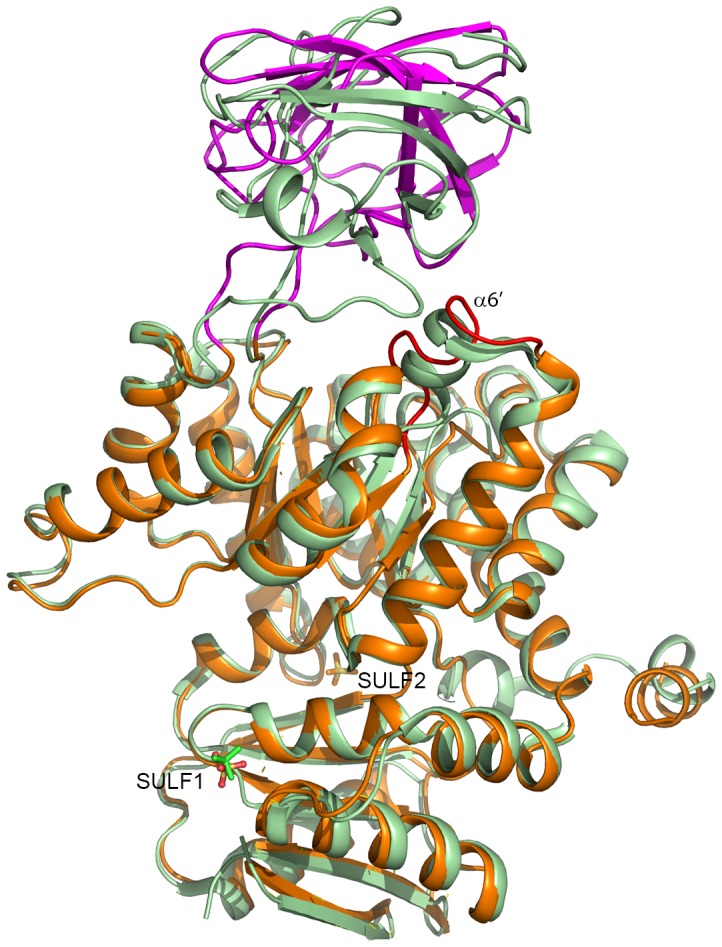
Cartoon drawing showing superposition of *C. parvum* pyruvate kinase structures. Our structure is orange, except for the B domain, which is magenta; 3MA8 is light green. Sulfate ions are shown as stick models (our structure - yellow and red; 3MA8 - green). The α6’ helix in our structure is highlighted in red.

As expected, the structure of CpPyK described here is very similar to the structure deposited in the PDB (3MA8) by the structural genomics consortium. The two protein samples differ in the sequence of the tag attached at the N-terminus. Crystallization conditions reported for 3MA8 were also significantly different from the conditions in which we crystallized CpPyK. While crystals for 3MA8 were obtained at 20C° using polyethylene glycol 3350 at pH 7.5, crystals used in our study were grown at 4°C using ammonium sulfate as precipitant at a much lower pH (4.0). As in our structure there are two molecules in the asymmetric unit of 3MA8. The packing of the dimers is similar in both structures, and the r.m.s.d. for the dimers is 0.95 Å. As expected, the individual monomers in these two structures have very similar overall structure (r. m. s. d. 0.65 Å for A monomers) except for the orientation of the N-helices and the B-domains (Fig. 7). Only a short stretch of N-helix was observed in our structure, and there is continuous electron density connecting the helix in each monomer to the side chain of Cys312 of the other (Figure S3). In 3MA8 the helix is joined to the A-domain, and no disulfide bond was modeled. However, the major difference between these structures is the orientation of the B-domain of each monomer with respect to the corresponding A-domain; thus, the r.m.s.d. between the A monomers of these structures (after removal of the B-domains) is reduced to 0.57 Å. The only sulfate ion modeled in the 3MA8 structure superimposes with SULF1 in the effector site of our structure (Fig. 7). Interestingly, the α6’ helices in both monomers of 3MA8 are intact, although the active site is unoccupied. Therefore, at least some of the differences between these two structures may have resulted from the difference in crystallization conditions or the presence of a glycerol molecule in the active site of our structure or a combination of both.

Further biochemical studies are needed to evaluate the regulatory mechanisms of Cryptosporidium PyK. The crystal structure reported here does not reveal any obvious difference in the active site of CpPyK as compared to the human enzyme. Additional biochemical and structural analyses of the parasitic protein will be necessary to identify mechanistic and structural features that may be targets for drug development.

## Materials and Methods

### Expression and Purification

Expression, purification and crystallization of CpPyK have been described previously [32]. Briefly, the coding sequence for CpPyK [13] was cloned into the *BamHI*/*HindIII* restriction sites in pET21a vector (Novagen). The reported primary sequence (Q5CSM7) for CpPyK in the protein database (http://www.uniprot.org/uniprot/) contains 532 amino acids. However, based on alignment with PyK sequences from various organisms using CLUSTALW [33], [34] we assigned the methionine residue at position 7 as the first residue of CpPyK. The recombinant CpPyK used in this study contained residues 2–526, a vector-derived T7 tag (MASMTGGQQMG) and three additional residues (RSG) at the N-terminus.

Recombinant CpPyK was expressed in *E. coli* BL21(DE3) cells in LB medium containing 50 µg/ml ampicillin and 0.2% glucose; induction was initiated with 0.4 mM isopropyl thio-β-galactopyranoside when the optical density of the culture reached 0.7–0.8, and the culture was grown overnight at 22°C. For purification, the frozen cell pellet was suspended in lysis buffer (50 mM Tris HCl, 0.1 M sodium chloride, 10 mM DTT, 0.1 mM phenylmethyl sulfonylfluoride, 1 mM benzamidine hydrochloride and 0.1 mg/ml lysozyme) and incubated at 4°C for 1 hr. The suspension was treated with DNAase I (1 µg/ml final) and subjected to centrifugation at 20,000 rpm for 30 min. Solid ammonium sulfate was added slowly to the clear supernatant with stirring; the pellet resulting from 25–40% ammonium sulfate saturation was suspended in 50 mM Tris HCl, 2.5 mM β-mercaptoethanol (BME), and 1 mM benzamidine hydrochloride (pH 8.2) and dialyzed overnight at 4°C against the same buffer. Recombinant CpPyK was further purified from the dialysate by anion exchange chromatography on a DEAE Sephacel column and eluted using a linear gradient of sodium chloride (0–0.5 M). Fractions containing CpPyK were pooled, concentrated and subjected to size exclusion chromatography on a preparative Superdex200 column equilibrated with 50 mM Tris HCl, 0.1 M sodium chloride, and 2.5 mM BME, pH 8.2. Fractions containing purified CpPyK eluted in a peak. Previously, we reported that the oligomeric state of CpPyK could not be ascertained from the results of size exclusion chromatography [32]. We subjected a partially purified preparation of CpPyK and catalase (Mr 232 KDa) to size exclusion chromatography separately on an analytical sizing column, Superdex 200 10/30 (GE life sciences). As shown in supplementary Fig. S1, the elution volumes were comparable.

Using a coupled enzyme assay we confirmed that recombinant CpPyK used for crystallization was enzymatically active [37]. In this assay *P. falciparum* lactate dehydrogenase (*Pf*LDH) was used to couple the oxidation of pyruvic acid generated in the reaction catalyzed by CpPyK. CpPyK activity was measured in the pH range 5.5–7.5. Measurement of CpPyK activity at lower pH was technically difficult, because activity of *Pf*LDH significantly dropped at lower pH. A typical assay mixture contained 2.5 mM phosphoenol pyruvate, 1 mM ADP, 5 mM magnesium chloride, 10 mM KCl, 0.2 mM NADH, 1 µg CpPyK and 0.18 µg *Pf*LDH in 50 mM HEPES buffer, pH 7.0. The rate of decrease in absorbance at 340 nm was followed for 1 min at 22°C in a UV spectrophotometer (Beckman Coulter DU640) (Fig. S1A). Enzyme activity was also measured in the presence of 1, 2 and 5 mM DTT and in the presence of 1 mM TCEP (Fig. S1B).

### Crystallization and Data Collection

Conditions for growing large single crystals were identified in a limited screening effort using only 196 conditions at 4°C and 22°C. Plate-shaped crystals exceeding 1 mm in the longest dimension were grown by the hanging drop vapor diffusion technique at 4°C using 0.4–0.8 M ammonium sulfate and 0.1 M sodium acetate buffer; the protein concentration was 7 mg/ml. Typically, these crystals grew at highly acidic pH ranging from 3.8 to 4.2 and reached maximum size in a week. The crystal used for structure analysis was grown at pH 4.0 with 0.65 M ammonium sulfate. Crystals were also grown by pre-incubating CpPyK with a non-hydrolyzable ATP analog (adenyl-5'-yl imidodiphosphate, 3 mM), 5 mM magnesium chloride and 5 mM pyruvic acid.

X-ray diffraction data were collected at SBC 19BM beam line at the Advanced Photon Source synchrotron facility. For data collection, the crystal was soaked in a cryoprotecting solution containing 25% glycerol (v/v) in the reservoir solution for approximately 5 min at 4°C and then placed in a nitrogen stream maintained at 100K. A total of 360 images was collected on a 210×210 mm^2^ CCD detector (MAR Research) at a crystal-to-detector distance of 200 mm, with 20 s exposure for each 0.5° oscillation frame. Intensity data were processed using the program package HKL2000 [38]. A crystal grown in the presence of ATP analog and pyruvic acid was also used for data collection. This crystal diffracted to ∼2.8 Å resolution and was isomorphous with the apo-form. However, the crystal suffered severe radiation damage, and a data set could not be collected.

### Structure Determination and Refinement

The structure of CpPyK was solved using the molecular replacement routines in CNS [39], using search models based on the *L. mexicana* PyK structure (1PKL) [20]. For the cross rotation search, three models were tested: the entire monomer (residues 1–479), a model containing domains A and C (residues 1–73 and 175–479), and domain B (residues 74–174). The entire monomer and the model containing domains A and C gave solutions with strong single peaks in the cross rotation, but no solution was obtained with domain B. The solution using only domains A and C was clearly better; the highest RF-function value was 0.0691, while the next highest value was only 0.0432 (corresponding values for the entire monomer were 0.0653 and 0.524, respectively). Translation functions were calculated with both solutions using data from 15 to 4 Å. The highest translation function T value for the model containing domains A and C was 0.322, while the T value for the entire monomer was only 0.209. Therefore, the model containing domains A and C was subjected to rigid body and then simulated annealing refinement, and 2F_o_–F_c_ maps were calculated to identify the location of domain B. It was clear that domain B had moved as a rigid body, and the density was of sufficient quality to allow placement of the entire domain into the partial model. Multiple cycles of refinement and rebuilding allowed replacement of non-identical residues and rebuilding of several regions where there were deletions or insertions compared to the *L. mexicana* structure. The graphics program Coot was used for model-building [40]. Residues 1–22, 33–41 and 508–517 could not be modeled due to very weak electron density in these areas.

Refinement of the structure was performed by simulated annealing using CNS with the stereochemical parameter files defined by Engh and Huber [41]. No sigma cutoff was applied to the data. Five percent of the data were randomly selected and removed prior to refinement for analysis of the free R factor. The two subunits in the asymmetric unit were restrained by the non-crystallographic symmetry throughout the simulated annealing refinement. As the refinement progressed, water molecules were added by using the water-picking routine in CNS, which searched the peaks in the 2F_o_–F_c_ map using a 3 sigma cutoff for density and checked distance criteria for reasonable hydrogen-bond donors and acceptors. All water molecules were subsequently verified by inspection of the maps. In the final stage of refinement we removed the noncrystallographic symmetry restraints and used the translation/libration/screw (TLS) [42] and restrained refinement option in REFMAC5 [43]. TLS parameters were generated using the TLS Motion Determination (TLSMD) server (http://skuld.bmsc.washington.edu/~tlsmd/) [44], [45]. Validation of the final model with MolProbity [46] produced a Clash Score of 8.87 (98th percentile for 271 structures in the resolution range 2.50±0.25 Å) and an overall score of 1.66 (99th percentile for 6960 structures in the same resolution range). Atomic coordinates and structure factors for CpPyK have been deposited in the Protein Data Bank (PDBID:4DRS). Fig. 3 was prepared using ESPript [47]; all other figures were prepared using Pymol [48].

## Supporting Information

Figure S1 **Characterization of CpPyK.** (A) Chromatogram showing elution of catalase (Mr 232 kDa) from Superdex 200 10/30 column. The flow rate was 0.5 ml/min, and fractions of 2 ml were collected. (B) Chromatogram showing elution of CpPyK from the same column at the same flow rate and fraction size. (C) SDS PAGE electrophoresis pattern of fractions collected in S1B. Twenty microliters of fractions 1–12 were boiled with an equal volume of 2X SDS sample denaturing buffer, and 10 µl mixtures were subjected to electrophoresis on 12% polyacrylamide gel containing 1% SDS. Lanes are labeled with the corresponding fraction number. The lane labeled M shows the standards with respective molecular weights shown in kDa. The gel was stained with Coomassie Blue.
(TIF)

Figure S2 **Analysis of enzymatic activity of CpPyK.** (A) CpPyK activity was measured by monitoring absorbance at 340 nm for 1 min at 22°C. Reactions were performed in 1 ml of 50 mM HEPES buffer, pH 7.0. (B) The effect of reducing agents on CpPyK activity was determined by incubating the enzyme in the presence of reducing agents (1, 2 and 5 mM DTT or 1 mM TCEP). Reaction velocities calculated as the rate of oxidation of NADH are shown along the Y-axis.
(TIF)

Figure S3 **Electron density maps showing the disulfide bonds in CpPyK.** All residues (23–32) in the N-helix of each monomer were truncated to alanine except residue 26, which was truncated to glycine, and the model was refined using Refmac5 [43]. Refined coordinates were used for calculating 2F_o_–F_c_ (magenta) and F_o_-F_c_ (yellow) electron density maps, which were displayed using Coot [40]. (A, B) Residues 26 and 29 of one monomer and residue 312 of the other monomer are labeled. The 2F_o_–F_c_ map is contoured at 1σ, and the F_o_-F_c_ map is contoured at 4.5σ level. Large residual electron density peaks are observed in locations occupied by the sulfur atoms of Cys26 and Met29 in our model.
(TIF)
