# Supplementary figures and images for: Crystal Structure of Cryptosporidium parvum Pyruvate Kinase
Source: PLoS One. 2012 Oct 9;7(10):e46875. doi: 10.1371/journal.pone.0046875 (PMC3467265; doi:10.1371/journal.pone.0046875)

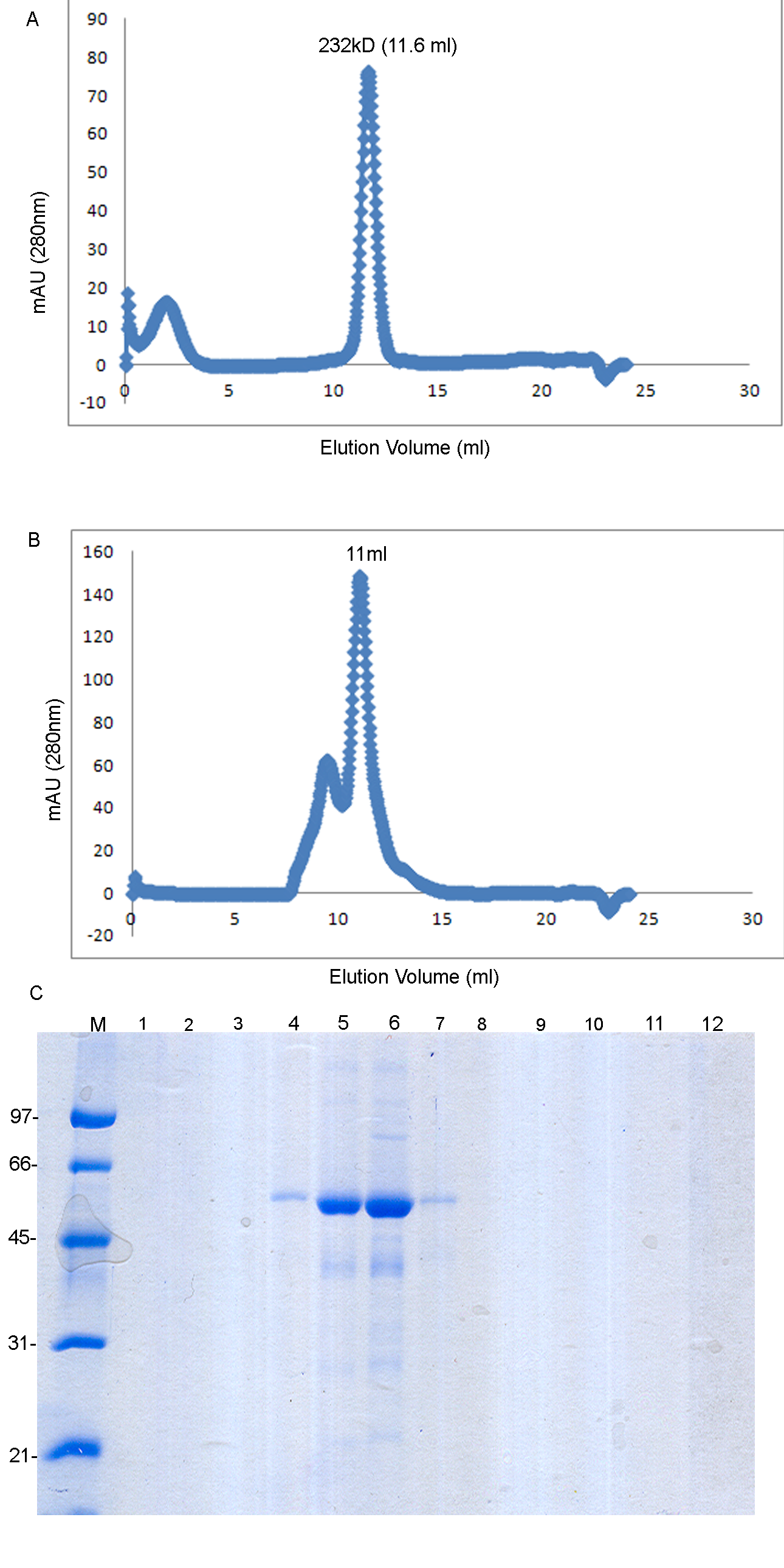

Supplement: Figure S1 — Characterization of CpPyK. (A) Chromatogram showing elution of catalase (Mr 232 kDa) from Superdex 200 10/30 column. The flow rate was 0.5 ml/min, and fractions of 2 ml were collected. (B) Chromatogram showing elution of CpPyK from the same column at the same flow rate and fraction size. (C) SDS PAGE electrophoresis pattern of fractions collected in S1B. Twenty microliters of fractions 1–12 were boiled with an equal volume of 2X SDS sample denaturing buffer, and 10 µl mixtures were subjected to electrophoresis on 12% polyacrylamide gel containing 1% SDS. Lanes are labeled with the corresponding fraction number. The lane labeled M shows the standards with respective molecular weights shown in kDa. The gel was stained with Coomassie Blue. (TIF) [file pone.0046875.s001.tif]

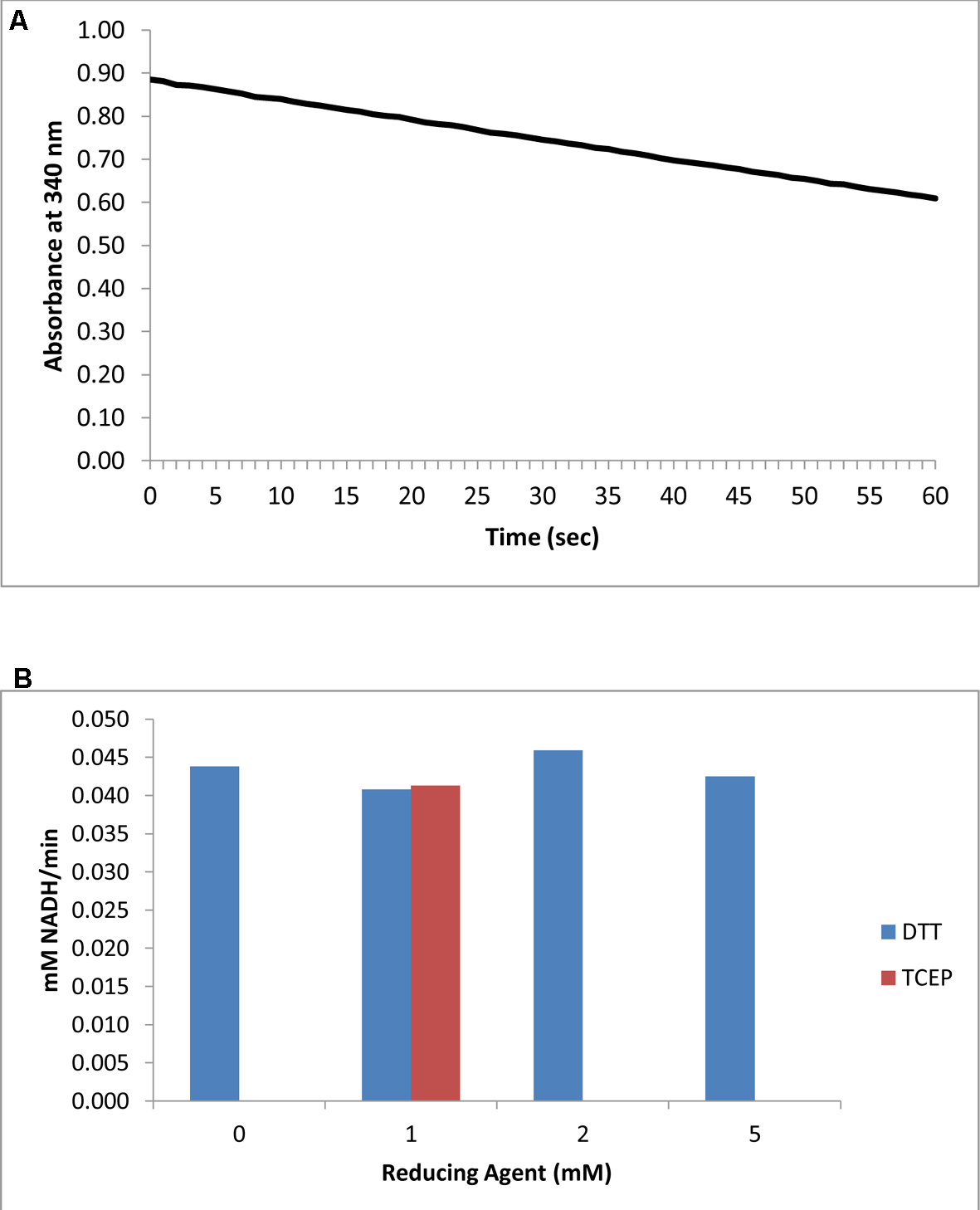

Supplement: Figure S2 — Analysis of enzymatic activity of CpPyK. (A) CpPyK activity was measured by monitoring absorbance at 340 nm for 1 min at 22°C. Reactions were performed in 1 ml of 50 mM HEPES buffer, pH 7.0. (B) The effect of reducing agents on CpPyK activity was determined by incubating the enzyme in the presence of reducing agents (1, 2 and 5 mM DTT or 1 mM TCEP). Reaction velocities calculated as the rate of oxidation of NADH are shown along the Y-axis. (TIF) [file pone.0046875.s002.tif]

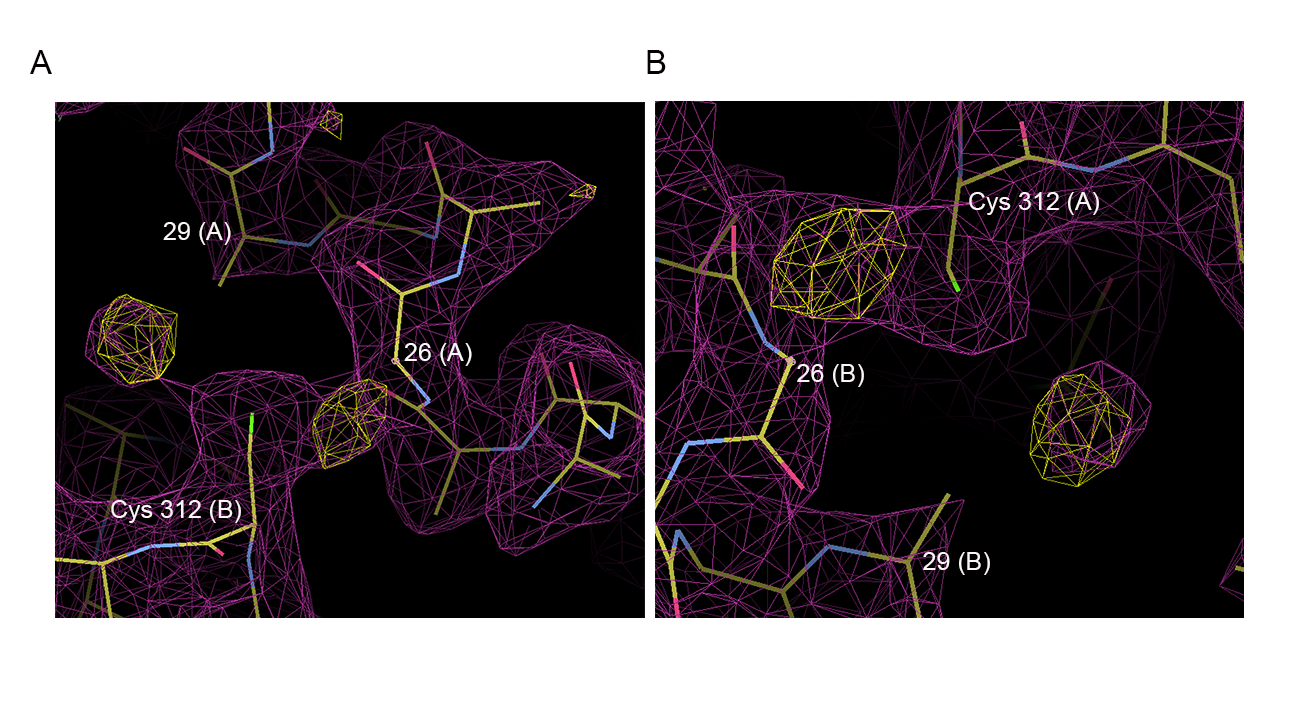

Supplement: Figure S3 — Electron density maps showing the disulfide bonds in CpPyK. All residues (23–32) in the N-helix of each monomer were truncated to alanine except residue 26, which was truncated to glycine, and the model was refined using Refmac5 [43]. Refined coordinates were used for calculating 2Fo–Fc (magenta) and Fo-Fc (yellow) electron density maps, which were displayed using Coot [40]. (A, B) Residues 26 and 29 of one monomer and residue 312 of the other monomer are labeled. The 2Fo–Fc map is contoured at 1σ, and the Fo-Fc map is contoured at 4.5σ level. Large residual electron density peaks are observed in locations occupied by the sulfur atoms of Cys26 and Met29 in our model. (TIF) [file pone.0046875.s003.tif]
